# Checking Robustness Between Weak Transactional Consistency Models

**DOI:** 10.1007/978-3-030-72019-3_4

**Published:** 2021-03-23

**Authors:** Sidi Mohamed Beillahi, Ahmed Bouajjani, Constantin Enea

**Affiliations:** Imperial College, London, UK; Université de Paris, IRIF, CNRS, Paris, France

**Keywords:** Transactional databases, Weak consistency, Program verification

## Abstract

Concurrent accesses to databases are typically encapsulated in transactions in order to enable isolation from other concurrent computations and resilience to failures. Modern databases provide transactions with various semantics corresponding to different trade-offs between consistency and availability. Since a weaker consistency model provides better performance, an important issue is investigating the weakest level of consistency needed by a given program (to satisfy its specification). As a way of dealing with this issue, we investigate the problem of checking whether a given program has the same set of behaviors when replacing a consistency model with a weaker one. This property known as *robustness* generally implies that any specification of the program is preserved when weakening the consistency. We focus on the robustness problem for consistency models which are weaker than standard serializability, namely, causal consistency, prefix consistency, and snapshot isolation. We show that checking robustness between these models is polynomial time reducible to a state reachability problem under serializability. We use this reduction to also derive a pragmatic proof technique based on Lipton’s reduction theory that allows to prove programs robust. We have applied our techniques to several challenging applications drawn from the literature of distributed systems and databases.

## 1 Introduction

Concurrent accesses to databases are typically encapsulated in transactions in order to enable isolation from other concurrent computations and resilience to failures. Modern databases provide transactions with various semantics corresponding to different tradeoffs between consistency and availability. The strongest consistency level is achieved with *serializable* transactions [42] whose outcome in concurrent executions is the same as if the transactions were executed atomically in some order. Since serializability (SER) carries a significant penalty on availability, modern databases often provide weaker consistency models, e.g.,

* This work is supported in part by the European Research Council (ERC) under the European Union's Horizon 2020 research and innovation programme (grant agreement No 678177).

causal consistency (CC) [38], prefix consistency (PC) [22, 25], and snapshot isolation (SI) [12]. Causal consistency requires that if a transaction $t_1$ "affects" another transaction $t_2$, e.g., $t_1$ executes before $t_2$ in the same session or $t_2$ reads a value written by $t_1$, then the updates in these two transactions are observed by any other transaction in this order. Concurrent transactions, which are not causally related to each other, can be observed in different orders, leading to behaviors that are not possible under SER. Prefix consistency requires that there is a total commit order between all the transactions such that each transaction observes all the updates in a prefix of this sequence (PC is stronger than CC). Two transactions can observe the *same* prefix, which leads to behaviors that are not admitted by SER. Snapshot isolation further requires that two different transactions observe different prefixes if they both write to a common variable.

Since a weaker consistency model provides better performance, an important issue is identifying the *weakest* level of consistency needed by a program (to satisfy its specification). One way to tackle this issue is checking whether a program $P$ designed under a consistency model $S$ has the same behaviors when run under a weaker consistency model $W$. This property of a program is generally known as *robustness* against substituting $S$ with $W$. It implies that any specification of $P$ is preserved when weakening the consistency model (from $S$ to $W$). Preserving any specification is convenient since specifications are rarely present in practice.

The problem of checking robustness for a given program has been investigated in several recent works, but only when the stronger model ($S$) is SER, e.g., [9, 10, 19, 26, 13, 40], or sequential consistency in the non-transactional case, e.g. [36, 15, 29]. However, there is a large class of specifications that can be implemented even in the presence of "anomalies", i.e., behaviors which are not admitted under SER (see [46] for a discussion). In this context, an important question is whether a certain implementation (program) is robust against substituting a weak consistency model, e.g., SI, with a weaker one, e.g., CC.

In this paper, we consider the sequence of increasingly strong consistency models mentioned above, CC, PC, and SI, and investigate the problem of checking robustness for a given program against weakening the consistency model to one in this range. We study the asymptotic complexity of this problem and propose effective techniques for establishing robustness based on abstraction. There are two important cases to consider: robustness against substituting SI with PC and PC with CC, respectively. Robustness against substituting SI with CC can be obtained as the conjunction of these two cases.

In the first case (SI vs PC), checking robustness for a program $P$ is reduced to a reachability (assertion checking) problem in a composition of $P$ under PC with a monitor that checks whether a PC behavior is an "anomaly", i.e., admitted by $P$ under PC, but not under SI. This approach raises two non-trivial challenges: (1) defining a monitor for detecting PC vs SI anomalies that uses a minimal amount of auxiliary memory (to remember past events), and (2) determining the complexity of checking if the composition of $P$ with the monitor reaches a specific control location[1] under the (weaker) model PC. Interestingly enough,

[1] We assume that the monitor goes to an error location when detecting an anomaly.

we address these two challenges by studying the relationship between these two weak consistency models, PC and SI, and *serializability.* The construction of the monitor is based on the fact that the PC vs SI anomalies can be defined as roughly, the difference between the PC vs SER and SI vs SER anomalies (investigated in previous work [13]), and we show that the reachability problem under PC can be reduced to a reachability problem under SER. These results lead to a polynomial-time reduction of this robustness problem (for arbitrary programs) to a reachability problem under SER, which is important from a practical point of view since the SER semantics (as opposed to the PC or SI semantics) can be encoded easily in existing verification tools (using locks to guard the isolation of transactions). These results also enable a precise characterization of the complexity class of this problem.

Checking robustness against substituting PC with CC is reduced to the problem of checking robustness against substituting SER with CC. The latter has been shown to be polynomial-time reducible to reachability under SER in [10]. This surprising result relies on the reduction from PC reachability to SER reachability mentioned above. This reduction shows that a given program $P$ reaches a certain control location under PC iff a transformed program $P'$, where essentially, each transaction is split in two parts, one part containing all the reads, and one part containing all the writes, reaches the same control location under SER. Since this reduction preserves the structure of the program, CC vs PC anomalies of a program $P$ correspond to CC vs SER anomalies of the transformed program $P'$.

Beyond enabling these reductions, the characterization of classes of anomalies or the reduction from the PC semantics to the SER semantics are also important for a better understanding of these weak consistency models and the differences between them. We believe that these results can find applications beyond robustness checking, e.g., verifying conformance to given specifications.

As a more pragmatic approach for establishing robustness, which avoids a non-reachability proof under SER, we have introduced a proof methodology that builds on Lipton's reduction theory [39] and the concept of commutativity dependency graph introduced in [9], which represents mover type dependencies between the transactions in a program. We give sufficient conditions for robustness in all the cases mentioned above, which characterize the commutativity dependency graph associated to a given program.

We tested the applicability of these verification techniques on a benchmark containing seven challenging applications extracted from previous work [30, 34, 19]. These techniques are precise enough for proving or disproving the robustness of all these applications, for all combinations of the consistency models.

Complete proofs and more details can be found in [11].

## 2 Overview

We give an overview of the robustness problems investigated in this paper, discussing first the case PC vs. CC, and then SI vs PC. We end with an example that illustrates the robustness checking technique based on commutativity arguments.

```
Process 1

CreateEvent(v, e1, 3):
[ Tickets[v][e1] := 3 ]

CountTickets(v):
[ r := ∑_e Tickets[v][e] ]
```

```
Process 2

CreateEvent(v, e2, 3):
[ Tickets[v][e2] := 3 ]

CountTickets(v):
[ r := ∑_e Tickets[v][e] ]
```

(a) FusionTicket.

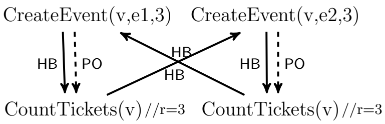

(b) A CC trace of FusionTicket.

```
Process 1

Register(u, p1):
[ r := RegisteredUsers[u]
  assume r == 0
  RegisteredUsers[u] := 1
  Password[u] := p1 ]
```

```
Process 2

Register(u, p2):
[ r := RegisteredUsers[u]
  assume r == 0
  RegisteredUsers[u] := 1
  Password[u] := p2 ]
```

(c) Twitter.

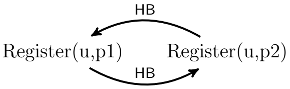

(d) A CC and PC trace of Twitter.

```
Process 1

RegisterRd(u, p1):
[ r := RegisteredUsers[u]
  assume r == 0 ]

RegisterWr(u, p1):
[ RegisteredUsers[u] := 1
  Password[u] := p1 ]
```

```
Process 2

RegisterRd(u, p2):
[ r := RegisteredUsers[u]
  assume r == 0 ]

RegisterWr(u, p2):
[ RegisteredUsers[u] := 1
  Password[u] := p2 ]
```

(e) Transformed Twitter.

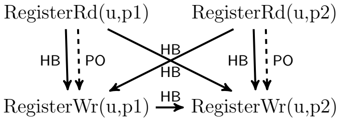

(f) A CC and SER trace of transformed Twitter.

```
Process 1

PlaceBet(1,2):
[ assume time < TIMEOUT
    Bets[1] := 2 ]
```

```
Process 2

PlaceBet(2,3):
[ assume time < TIMEOUT
    Bets[2] := 3 ]
```

```
Process 3

SettleBet():
[Bets' := Bets
 n := Bets'.Length
 assume time > TIMEOUT & n > 0
 select i s.t. Bets'[i] ≠ ⊥
 return := Bets'[i] ]
```

(g) Betting.

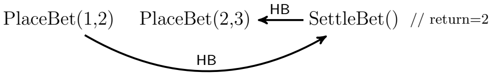

(h) A PC and SI trace of Betting.

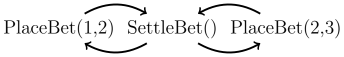

(i) Commutativity dependency graph of Betting.

Fig. 1: Transactional programs and traces under different consistency models.

**Robustness PC vs CC.** We illustrate the robustness against substituting PC with CC using the FusionTicket and the Twitter programs in Figure 1a and Figure 1c, respectively. FusionTicket manages tickets for a number of events, each event being associated with a venue. Its state consists of a two-dimensional map that stores the number of tickets for an event in a given venue ($r$ is a local variable, and the assignment in `CountTickets` is interpreted as a read of the shared state). The program has two processes and each process contains two transactions. The first transaction creates an event e in a venue v with a number of tickets n, and the second transaction computes the total number of tickets for all the events in a venue v. A possible candidate for a specification of this program is that the values computed in `CountTickets` are monotonically increasing since each such value is computed after creating a new event. Twitter provides a transaction for registering a new user with a given username and password, which is executed by two parallel processes. Its state contains two maps that record whether a given username has been registered (0 and 1 stand for non-registered and registered, respectively) and the password for a given username. Each transaction first checks whether a given username is free (see the `assume` statement). The intended specification is that the user must be registered with the given password when the registration transaction succeeds.

A program is robust against substituting `PC` with `CC` if its set of behaviors under the two models coincide. We model behaviors of a given program as *traces*, which record standard control-flow and data-flow dependencies between transactions, e.g., the order between transactions in the same session and whether a transaction reads the value written by another (read-from). The transitive closure of the union of all these dependency relations is called *happens-before*. Figure 1b pictures a trace of FusionTicket where the concrete values which are read in a transaction are written under comments. In this trace, each process registers a different event but in the same venue and with the same number of tickets, and it ignores the event created by the other process when computing the sum of tickets in the venue.

Figure 1b pictures a trace of FusionTicket under `CC`, which is a witness that FusionTicket is *not* robust against substituting `PC` with `CC`. This trace is also a violation of the intended specification since the number of tickets is not increasing (the sum of tickets is 3 in both processes). The happens-before dependencies (pictured with `HB` labeled edges) include the program-order `PO` (the order between transactions in the same process), and read-write dependencies, since an instance of CountTickets(v) does not observe the value written by the CreateEvent transaction in the other process (the latter overwrites some value that the former reads). This trace is allowed under `CC` because the transaction CreateEvent(v, e1, 3) executes concurrently with the transaction CountTickets(v) in the other process, and similarly for CreateEvent(v, e2, 3). However, it is not allowed under `PC` since it is impossible to define a total commit order between CreateEvent(v, e1, 3) and CreateEvent(v, e2, 3) that justifies the reads of both CountTickets(v) transactions (these reads should correspond to the updates in a prefix of this order). For instance, assuming that CreateEvent(v, e1, 3) commits before CreateEvent(v, e2, 3), CountTickets(v) in the second process must observe the effect of CreateEvent(v, e1, 3) as well since it observes the effect of CreateEvent(v, e2, 3). However, this contradicts the fact that CountTickets(v) computes the sum of tickets as being 3.

On the other hand, Twitter is robust against substituting `PC` with `CC`. For instance, Figure 1d pictures a trace of Twitter under `CC`, where the `assume` in both transactions pass. In this trace, the transactions Register(u,p1) and Register(u,p2) execute concurrently and are unaware of each other's writes (they are not causally related). The `HB` dependencies include write-write dependencies since both transactions write on the same location (we consider the transaction in Process 2 to be the last one writing to the `Password` map), and read-write dependencies since each transaction reads `RegisteredUsers` that is written by the other. This trace is also allowed under `PC` since the commit order can be defined such that Register(u,p1) is ordered before Register(u,p2), and then both transactions read from the initial state (the empty prefix). Note that this trace has a cyclic happens-before which means that it is not allowed under serializability.

**Checking robustness `PC` vs `CC`.** We reduce the problem of checking robustness against substituting `PC` with `CC` to the robustness problem against substituting `SER` with `CC` (the latter reduces to a reachability problem under `SER` [10]). This reduction relies on a syntactic program transformation that rewrites `PC` behaviors of a given program $P$ to `SER` behaviors of another program $P'$. The program $P'$ is obtained by splitting each transaction $t$ of $P$ into two transactions: the first transaction performs all the reads in $t$ and the second performs all the writes in $t$ (the two are related by program order). Figure 1e shows this transformation applied on Twitter. The trace in Figure 1f is a serializable execution of the transformed Twitter which is "observationally" equivalent to the trace in Figure 1d of the original Twitter, i.e., each read of the shared state returns the same value and the writes on the shared state are applied in the same order (the acyclicity of the happens-before shows that this is a serializable trace). The transformed FusionTicket coincides with the original version because it contains no transaction that both reads and writes on the shared state.

We show that `PC` behaviors and `SER` behaviors of the original and transformed program, respectively, are related by a bijection. In particular, we show that any `PC` vs. `CC` robustness violation of the original program manifests as a `SER` vs. `CC` robustness violation of the transformed program, and vice-versa. For instance, the `CC` trace of the original Twitter in Figure 1d corresponds to the `CC` trace of the transformed Twitter in Figure 1f, and the acyclicity of the latter (the fact that it is admitted by `SER`) implies that the former is admitted by the original Twitter under `PC`. On the other hand, the trace in Figure 1b is also a `CC` of the transformed FusionTicket and its cyclicity implies that it is not admitted by FusionTicket under `PC`, and thus, it represents a robustness violation.

**Robustness `SI` vs `PC`.** We illustrate the robustness against substituting `SI` with `PC` using Twitter and the Betting program in Figure 1g. Twitter is *not* robust against substituting `SI` with `PC`, the trace in Figure 1d being a witness violation. This trace is also a violation of the intended specification since one of the users registers a password that is overwritten in a concurrent transaction. This `PC` trace is not possible under `SI` because Register(u,p1) and Register(u,p2) observe the same prefix of the commit order (i.e., an empty prefix), but they write to a common memory location Password[u] which is not allowed under `SI`.

On the other hand, the Betting program in Figure 1g, which manages a set of bets, is robust against substituting `SI` with `PC`. The first two processes execute one transaction that places a bet of a value v with a unique bet identifier id, assuming that the bet expiration time is not yet reached (bets are recorded in the map `Bets`). The third process contains a single transaction that settles the betting assuming that the bet expiration time was reached and at least one bet has been placed. This transaction starts by taking a snapshot of the `Bets` map into a local variable `Bets'`, and then selects a random non-null value (different from $\perp$) in the map to correspond to the winning bet. The intended specification of this program is that the winning bet corresponds to a genuine bet that was placed. Figure 1g pictures a `PC` trace of Betting where SettleBet observes only the bet of the first process PlaceBet(1,2). The `HB` dependency towards the second process denotes a read-write dependency (SettleBet reads a cell of the map `Bets` which is overwritten by the second process). This trace is allowed under `SI` because no two transactions write to the same location.

**Checking robustness `SI` vs `PC`.** We reduce robustness against substituting `PC` with `CC` to a reachability problem under `SER`. This reduction is based on a characterization of happens-before cycles[2] that are possible under `PC` but not `SI`, and the transformation described above that allows to simulate the `PC` semantics of a program on top of `SER`. The former is used to define an instrumentation (monitor) for the transformed program that reaches an error state iff the original program is not robust. Therefore, we show that the happens-before cycles in `PC` traces that are not admitted by `SI` must contain a transaction that (1) overwrites a value written by another transaction in the cycle and (2) reads a value overwritten by another transaction in the cycle. For instance, the trace of Twitter in Figure 1d is not allowed under `SI` because Register(u,p2) overwrites a value written by Register(u,p1) (the password) and reads a value overwritten by Register(u,p1) (checking whether the username $u$ is registered). The trace of Betting in Figure 1g is allowed under `SI` because its happens-before is acyclic.

**Checking robustness using commutativity arguments.** Based on the reductions above, we propose an approximated method for proving robustness based on the concept of mover in Lipton's reduction theory [39]. A transaction is a left (resp., right) mover if it commutes to the left (resp., right) of another transaction (by a different process) while preserving the computation. We use the notion of mover to characterize the data-flow dependencies in the happens-before. Roughly, there exists a data-flow dependency between two transactions in some execution if one doesn't commute to the left/right of the other one.

We define a commutativity dependency graph which summarizes the happens-before dependencies in all executions of a transformed program (obtained by splitting the transactions of the original program as explained above), and derive a proof method for robustness which inspects paths in this graph. Two transactions $t_1$ and $t_2$ are linked by a directed edge iff $t_1$ *cannot* move to the right of $t_2$ (or $t_2$ cannot move to the left of $t_1$), or if they are related by the program order. Moreover, two transactions $t_1$ and $t_2$ are linked by an undirected edge iff they are the result of splitting the same transaction.

A program is robust against substituting `PC` with `CC` if roughly, its commutativity dependency graph does *not* contain a *simple* cycle of directed edges with two distinct transactions $t_1$ and $t_2$, such that $t_1$ does not commute left because of another transaction $t_3$ in the cycle that reads a variable that $t_1$ writes to,

[2] Traces with an acyclic happens-before are not robustness violations because they are admitted under serializability, which implies that they are admitted under the weaker model `SI` as well.

$\langle prog \rangle$ ::= `program` $\langle process \rangle^*$

$\langle process \rangle$ ::= `process` $\langle pid \rangle$ `regs` $\langle reg \rangle^*$ $\langle txn \rangle^*$

$\langle txn \rangle$ ::= `begin` $\langle read \rangle^*$ $\langle test \rangle^*$ $\langle write \rangle^*$ `commit`

$\langle read \rangle$ ::= $\langle label \rangle$`:` $\langle reg \rangle$ `:=` $\langle var \rangle$`;` `goto` $\langle label \rangle$`;`

$\langle test \rangle$ ::= $\langle label \rangle$`:` `assume` $\langle bexpr \rangle$`;` `goto` $\langle label \rangle$`;`

$\langle write \rangle$ ::= $\langle label \rangle$`:` $\langle var \rangle$ `:=` $\langle reg\text{-}expr \rangle$`;` `goto` $\langle label \rangle$`;`

Fig. 2: The syntax of our programming language. $a^*$ indicates zero or more occurrences of $a$. $\langle pid \rangle$, $\langle reg \rangle$, $\langle label \rangle$, and $\langle var \rangle$ represent a process identifier, a register, a label, and a shared variable, respectively. $\langle reg\text{-}expr \rangle$ is an expression over registers while $\langle bexpr \rangle$ is a Boolean expression over registers, or the non-deterministic choice $*$.

and $t_2$ does not commute right because of another transaction $t_4$ in the cycle ($t_3$ and $t_4$ can coincide) that writes to a variable that $t_2$ either reads from or writes to[3]. For instance, Figure 1i shows the commutativity dependency graph of the transformed Betting program, which coincides with the original Betting because PlaceBet(1,2) and PlaceBet(2,3) are write-only transactions and SettleBet() is a read-only transaction. Both simple cycles in Figure 1i contain just two transactions and therefore do not meet the criterion above which requires at least 3 transactions. Therefore, Betting is robust against substituting `PC` with `CC`.

A program is robust against substituting `SI` with `PC`, if roughly, its commutativity dependency graph does *not* contain a *simple* cycle with two successive transactions $t_1$ and $t_2$ that are linked by an undirected edge, such that $t_1$ does not commute left because of another transaction $t_3$ in the cycle that writes to a variable that $t_1$ writes to, and $t_2$ does not commute right because of another transaction $t_4$ in the cycle ($t_3$ and $t_4$ can coincide) that writes to a variable that $t_2$ reads from[4]. Betting is also robust against substituting `SI` with `PC` for the same reason (simple cycles of size 2).

## 3 Consistency Models

**Syntax.** We present our results in the context of the simple programming language, defined in Figure 2, where a program is a parallel composition of *processes* distinguished using a set of identifiers $\mathbb{P}$. A process is a sequence of *transactions* and each transaction is a sequence of *labeled instructions*. A transaction starts with a `begin` instruction and finishes with a `commit` instruction. Instructions include assignments to a process-local *register* from a set $\mathbb{R}$ or to a *shared variable* from a set $\mathbb{V}$, or an `assume`. The assignments use values from a data domain

[3] The transactions $t_1$, $t_2$, $t_3$, and $t_4$ correspond to $t_1$, $t_i$, $t_n$, and $t_{i+1}$, respectively, in Theorem 6.

[4] The transactions $t_1$, $t_2$, $t_3$, and $t_4$ correspond to $t_1$, $t_2$, $t_n$, and $t_3$, respectively, in Theorem 7.

$\mathbb{D}$. An assignment to a register $\langle reg \rangle := \langle var \rangle$ is called a *read* of the shared-variable $\langle var \rangle$ and an assignment to a shared variable $\langle var \rangle := \langle reg \rangle$ is called a *write* to the shared-variable $\langle var \rangle$. The `assume` $\langle bexpr \rangle$ blocks the process if the Boolean expression $\langle bexpr \rangle$ over registers is false. It can be used to model conditionals. The `goto` statement transfers the control to the program location (instruction) specified by a given label. Since multiple instructions can have the same label, `goto` statements can be used to mimic imperative constructs like loops and conditionals inside transactions.

We assume w.l.o.g. that every transaction is written as a sequence of reads or `assume` statements followed by a sequence of writes (a single `goto` statement from the sequence of read/`assume` instructions transfers the control to the sequence of writes). In the context of the consistency models we study in this paper, every program can be equivalently rewritten as a set of transactions of this form.

To simplify the technical exposition, programs contain a bounded number of processes and each process executes a bounded number of transactions. A transaction may execute an unbounded number of instructions but these instructions concern a bounded number of variables, which makes it impossible to model SQL (select/update) queries that may access tables with a statically unknown number of rows. Our results can be extended beyond these restrictions as explained in Remark 1 and Remark 2.

**Semantics.** We describe the semantics of a program under four consistency models, i.e., causal consistency[5] (CC), prefix consistency (PC), snapshot isolation (SI), and serializability (SER).

In the semantics of a program under CC, shared variables are replicated across each process, each process maintaining its own local valuation of these variables. During the execution of a transaction in a process, its writes are stored in a *transaction log* that can be accessed only by the process executing the transaction and that is broadcasted to all the other processes at the end of the transaction. To read a shared variable $x$, a process $p$ first accesses its transaction log and takes the last written value on $x$, if any, and then its own valuation of the shared variable, if $x$ was not written during the current transaction. Transaction logs are delivered to every process in an order consistent with the *causal* relation between transactions, i.e., the transitive closure of the union of the *program order* (the order in which transactions are executed by a process), and the *read-from* relation (a transaction $t_1$ reads-from a transaction $t_2$ iff $t_1$ reads a value that was written by $t_2$). When a process receives a transaction log, it immediately applies it on its shared-variable valuation.

In the semantics of a program under PC and SI, shared variables are stored in a central memory and each process keeps a local valuation of these variables. When a process starts a new transaction, it fetches a consistent snapshot of the shared variables from the central memory and stores it in its local valuation of these variables. During the execution of a transaction in a process, writes to shared variables are stored in the local valuation of these variables, and in a transaction log. To read a shared variable, a process takes its own valuation of the

[5] We consider a variation known as causal convergence [20, 16]

shared variable. A process commits a transaction by applying the updates in the transaction log on the central memory in an atomic way (to make them visible to all processes). Under SI, when a process applies the writes in a transaction log on the central memory, it must ensure that there were no concurrent writes that occurred after the last fetch from the central memory to a shared variable that was written during the current transaction. Otherwise, the transaction is aborted and its effects discarded.

In the semantics of a program under SER, we adopt a simple operational model where we keep a single shared-variable valuation in a central memory (accessed by all processes) with the standard interpretation of read and write statements. Transactions execute serially, one after another.

We use a standard model of executions of a program called *trace*. A trace represents the order between transactions in the same process, and the data-flow in an execution using standard happens-before relations between transactions. We assume that each transaction in a program is identified uniquely using a transaction identifier from a set $\mathbb{T}$. Also, $f : \mathbb{T} \rightarrow 2^{\mathbb{S}}$ is a mapping that associates each transaction in $\mathbb{T}$ with a sequence of read and write events from the set

$$\mathbb{S} = \{\mathsf{re}(t, x, v), \mathsf{we}(t, x, v) : t \in \mathbb{T}, x \in \mathbb{V}, v \in \mathbb{D}\}$$

where $\mathsf{re}(t, x, v)$ is a read of $x$ returning $v$, and $\mathsf{we}(t, x, v)$ is a write of $v$ to $x$.

**Definition 1.** *A* trace *is a tuple* $\tau = (\rho, f, \mathsf{TO}, \mathsf{PO}, \mathsf{WR}, \mathsf{WW}, \mathsf{RW})$ *where* $\rho \subseteq \mathbb{T}$ *is a set of transaction identifiers, and*

- $\mathsf{TO}$ *is a mapping giving the order between events in each transaction, i.e., it associates each transaction* $t$ *in* $\rho$ *with a total order* $\mathsf{TO}(t)$ *on* $f(t) \times f(t)$.
- $\mathsf{PO}$ *is the program order relation, a strict partial order on* $\rho \times \rho$ *that orders every two transactions issued by the same process.*
- $\mathsf{WR}$ *is the read-from relation between distinct transactions* $(t1, t2) \in \rho \times \rho$ *representing the fact that* $t2$ *reads a value written by* $t1$.
- $\mathsf{WW}$ *is the store order relation on* $\rho \times \rho$ *between distinct transactions that write to the same shared variable.*
- $\mathsf{RW}$ *is the conflict order relation between distinct transactions, defined by* $\mathsf{RW} = \mathsf{WR}^{-1}; \mathsf{WW}$ *(; denotes the sequential composition of two relations).*

For simplicity, for a trace $\tau = (\rho, f, \mathsf{TO}, \mathsf{PO}, \mathsf{WR}, \mathsf{WW}, \mathsf{RW})$, we write $t \in \tau$ instead of $t \in \rho$. We also assume that each trace contains a fictitious transaction that writes the initial values of all shared variables, and which is ordered before any other transaction in program order. Also, $\mathbb{T}\mathsf{r}_{\mathsf{X}}(\mathcal{P})$ is the set of traces representing executions of program $\mathcal{P}$ under a consistency model $\mathsf{X}$.

For each $\mathsf{X} \in \{\mathtt{CC}, \mathtt{PC}, \mathtt{SI}, \mathtt{SER}\}$, the set of traces $\mathbb{T}\mathsf{r}_{\mathsf{X}}(\mathcal{P})$ can be described using the set of properties in Table 1. A trace $\tau$ is possible under causal consistency iff there exist two relations $\mathsf{CO}$ a partial order (causal order) and $ARB$ a total order (arbitration order) that includes $\mathsf{CO}$, such that the properties $\mathsf{AxCausal}$, $\mathsf{AxArb}$, and $\mathsf{AxRetVal}$ hold [27, 16]. $\mathsf{AxCausal}$ guarantees that the program order and the read-from relation are included in the causal order, and $\mathsf{AxArb}$ guarantees

| | |
|---|---|
| AxCausal | $\mathsf{CO}_0^+ \subseteq \mathsf{CO}$ |
| AxArb | $ARB_0^+ \subseteq ARB$ |
| AxCC | AxRetVal $\wedge$ AxCausal $\wedge$ AxArb |
| AxPrefix | $ARB; \mathsf{CO} \subseteq \mathsf{CO}$ |
| AxPC | AxPrefix $\wedge$ AxCC |
| AxConflict | $\mathsf{WW} \subseteq \mathsf{CO}$ |
| AxSI | AxConflict $\wedge$ AxPC |
| AxSer | AxRetVal $\wedge$ AxCausal $\wedge$ AxArb $\wedge$ $\mathsf{CO} = ARB$ |

where
$\mathsf{CO}_0 = \mathsf{PO} \cup \mathsf{WR}$ and $ARB_0 = \mathsf{PO} \cup \mathsf{WR} \cup \mathsf{WW}$
AxRetVal $= \forall\, t \in \tau.\ \forall\ \mathsf{re}(t, x, v) \in f(t)$ we have that

- there exist a transaction $t_0 = Max_{ARB}(\{t' \in \tau \mid (t', t) \in \mathsf{CO} \wedge \exists\ \mathsf{we}(t', x, \cdot) \in f(t')\})$ and an event $\mathsf{we}(t_0, x, v) = Max_{\mathsf{TO}(t_0)}(\{\mathsf{we}(t_0, x, \cdot) \in f(t_0)\})$.

Table 1: Declarative definitions of consistency models. For an order relation $\leq$, $a = Max_{\leq}(A)$ iff $a \in A \wedge \forall\ b \in A.\ b \leq a$.

that the causal order and the store order are included in the arbitration order. AxRetVal guarantees that a read returns the value written by the last write in the last transaction that contains a write to the same variable and that is ordered by CO before the read's transaction. We use AxCC to denote the conjunction of these three properties. A trace $\tau$ is possible under prefix consistency iff there exist a causal order CO and an arbitration order $ARB$ such that AxCC holds and the property AxPrefix holds as well [27]. AxPrefix guarantees that every transaction observes a prefix of transactions that are ordered by $ARB$ before it. We use AxPC to denote the conjunction of AxCC and AxPrefix. A trace $\tau$ is possible under snapshot isolation iff there exist a causal order CO and an arbitration order $ARB$ such that AxPC holds and the property AxConflict holds [27]. AxConflict guarantees that if two transactions write to the same variable then one of them must observe the other. We use AxSI to denote the conjunction of AxPC and AxConflict. A trace $\tau$ is serializable iff there exist a causal order CO and an arbitration order $ARB$ such that the property AxSer holds which implies that the two relations CO and $ARB$ coincide. Note that for any given program $\mathcal{P}$, $\mathbb{T}\mathsf{r}_{\mathtt{SER}}(\mathcal{P}) \subseteq \mathbb{T}\mathsf{r}_{\mathtt{SI}}(\mathcal{P}) \subseteq \mathbb{T}\mathsf{r}_{\mathsf{PC}}(\mathcal{P}) \subseteq \mathbb{T}\mathsf{r}_{\mathsf{CC}}(\mathcal{P})$. Also, the four consistency models we consider disallow anomalies such as dirty and phantom reads.

For a given trace $\tau = (\rho, f, \mathsf{TO}, \mathsf{PO}, \mathsf{WR}, \mathsf{WW}, \mathsf{RW})$, the happens before order is the transitive closure of the union of all the relations in the trace, i.e., $\mathsf{HB} = (\mathsf{PO} \cup \mathsf{WR} \cup \mathsf{WW} \cup \mathsf{RW})^+$. A classic result states that a trace $\tau$ is serializable iff HB is acyclic [2, 47]. Note that HB is acyclic implies that WW is a total order between transactions that write to the same variable, and $(\mathsf{PO} \cup \mathsf{WR})^+$ and $(\mathsf{PO} \cup \mathsf{WR} \cup \mathsf{WW})^+$ are acyclic.

### 3.1 Robustness

In this work, we investigate the problem of checking whether a program $\mathcal{P}$ under a semantics $\mathsf{Y} \in \{\mathtt{PC},\ \mathtt{SI}\}$ produces the same set of traces as under a weaker semantics $\mathsf{X} \in \{\mathtt{CC},\ \mathtt{PC}\}$. When this holds, we say that $\mathcal{P}$ is *robust* against X relative to Y.

**Definition 2.** *A program $\mathcal{P}$ is called* robust *against a semantics $\mathsf{X} \in \{\texttt{CC}, \texttt{PC}, \texttt{SI}\}$ relative to a semantics $\mathsf{Y} \in \{\texttt{PC}, \texttt{SI}, \texttt{SER}\}$ such that $\mathsf{Y}$ is stronger than $\mathsf{X}$ iff $\mathbb{T}r_{\mathsf{X}}(\mathcal{P}) = \mathbb{T}r_{\mathsf{Y}}(\mathcal{P})$.*

If $\mathcal{P}$ is not robust against X relative to Y then there must exist a trace $\tau \in \mathbb{T}r_{\mathsf{X}}(\mathcal{P}) \setminus \mathbb{T}r_{\mathsf{Y}}(\mathcal{P})$. We say that $\tau$ is a robustness violation trace.

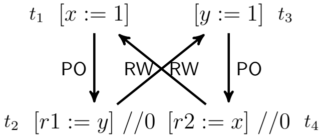

(a) Store Buffering (SB).

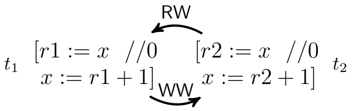

(b) Lost Update (LU).

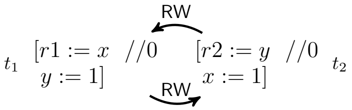

(c) Write Skew (WS).

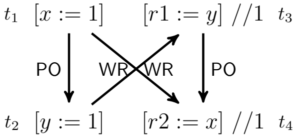

(d) Message Passing (MP).

Fig. 3: Litmus programs

We illustrate the notion of robustness on the programs in Figure 3, which are commonly used in the literature. In all programs, transactions of the same process are aligned vertically and ordered from top to bottom. Each read instruction is commented with the value it reads in some execution.

The store buffering (SB) program in Figure 3a contains four transactions that are issued by two distinct processes. We emphasize an execution where $t_2$ reads 0 from $y$ and $t_4$ reads 0 from $x$. This execution is allowed under CC since the two writes by $t_1$ and $t_3$ are not causally dependent. Thus, $t_2$ and $t_4$ are executed without seeing the writes from $t_3$ and $t_1$, respectively. However, this execution is not feasible under PC (which implies that it is not feasible under both SI and SER). In particular, we cannot have neither $(t_1, t_3) \in ARB$ nor $(t_3, t_1) \in ARB$ which contradicts the fact that $ARB$ is total order. For example, if $(t_1, t_3) \in ARB$, then $(t_1, t_4) \in \mathsf{CO}$ (since $ARB; \mathsf{CO} \subset \mathsf{CO}$) which contradicts the fact that $t_4$ does not see $t_1$. Similarly, $(t_3, t_1) \in ARB$ implies that $(t_3, t_2) \in \mathsf{CO}$ which contradicts the fact that $t_2$ does not see $t_3$. Thus, SB is not robust against CC relative to PC.

The lost update (LU) program in Figure 3b has two transactions that are issued by two distinct processes. We highlight an execution where both transactions read 0 from $x$. This execution is allowed under PC since both transactions are not causally dependent and can be executed in parallel by the two processes. However, it is not allowed under SI since both transactions write to a common variable (i.e., $x$). Thus, they cannot be executed in parallel and one of them must see the write of the other. Thus, SB is not robust against PC relative to SI.

The write skew (WS) program in Figure 3c has two transactions that are issued by two distinct processes. We highlight an execution where $t_1$ reads 0 from $x$ and $t_2$ reads 0 from $y$. This execution is allowed under SI since both transactions are not causally dependent, do not write to a common variable, and can be executed in parallel by the two processes. However, this execution is not allowed under SER since one of the two transactions must see the write of the other. Thus, WS is not robust against SI relative to SER.

The message passing (MP) program in Figure 3d has four transactions issued by two processes. Because $t_1$ and $t_2$ are causally dependent, under any semantics $\mathsf{X} \in \{\texttt{CC}, \texttt{PC}, \texttt{SI}, \texttt{SER}\}$ we only have three possible executions of MP, which correspond to either $t_3$ and $t_4$ not observing the writes of $t_1$ and $t_2$, or $t_3$ and $t_4$ observe the writes of both $t_1$ and $t_2$, or $t_4$ observes the write of $t_1$ (we highlight the values read in the second case in Figure 3d). Therefore, the executions of this program under the four consistency models coincide. Thus, MP is robust against CC relative to any other model.

## 4 Robustness Against CC Relative to PC

We show that checking robustness against CC relative to PC can be reduced to checking robustness against CC relative to SER. The crux of this reduction is a program transformation that allows to simulate the PC semantics of a program $\mathcal{P}$ using the SER semantics of a program $\mathcal{P}_\clubsuit$. Checking robustness against CC relative to SER can be reduced in polynomial time to reachability under SER [10].

Given a program $\mathcal{P}$ with a set of transactions $\mathsf{Tr}(\mathcal{P})$, we define a program $\mathcal{P}_\clubsuit$ such that every transaction $t \in \mathsf{Tr}(\mathcal{P})$ is split into a transaction $t[r]$ that contains all the read/`assume` statements in $t$ (in the same order) and another transaction $t[w]$ that contains all the write statements in $t$ (in the same order). In the following, we establish the following result:

**Theorem 1.** *A program $\mathcal{P}$ is robust against* CC *relative to* PC *iff $\mathcal{P}_\clubsuit$ is robust against* CC *relative to* SER*.*

Intuitively, under PC, processes can execute concurrent transactions that fetch the same consistent snapshot of the shared variables from the central memory and subsequently commit their writes. Decoupling the read part of a transaction from the write part allows to simulate such behaviors even under SER.

The proof of this theorem relies on several intermediate results concerning the relationship between traces of $\mathcal{P}$ and $\mathcal{P}_\clubsuit$. Let $\tau = (\rho, \mathsf{PO}, \mathsf{WR}, \mathsf{WW}, \mathsf{RW}) \in \mathbb{T}\mathsf{r}_\mathsf{X}(\mathcal{P})$ be a trace of a program $\mathcal{P}$ under a semantics X. We define the trace $\tau_\clubsuit = (\rho_\clubsuit, \mathsf{PO}_\clubsuit, \mathsf{WR}_\clubsuit, \mathsf{WW}_\clubsuit, \mathsf{RW}_\clubsuit)$ where every transaction $t \in \tau$ is split into two transactions $t[r] \in \tau_\clubsuit$ and $t[w] \in \tau_\clubsuit$, and the dependency relations are straightforward adaptations, i.e.,

- $\mathsf{PO}_\clubsuit$ is the smallest transitive relation that includes $(t[r], t[w])$ for every $t$, and $(t[w], t'[r])$ if $(t, t') \in \mathsf{PO}$,
- $(t'[w], t[r]) \in \mathsf{WR}_\clubsuit$, $(t'[w], t[w]) \in \mathsf{WW}_\clubsuit$, and $(t'[r], t[w]) \in \mathsf{RW}_\clubsuit$ if $(t', t) \in \mathsf{WR}$, $(t', t) \in \mathsf{WW}$, and $(t', t) \in \mathsf{RW}$, respectively.

For instance, Figure 4 pictures the trace $\tau_\clubsuit$ for the LU trace $\tau$ given in Figure 3b. For traces $\tau$ of programs that contain singleton transactions, e.g., SB in Figure 3a, $\tau_\clubsuit$ coincides with $\tau$.

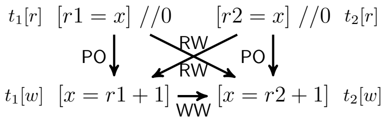

Fig. 4: A trace of the transformed LU program ($\mathsf{LU}_\clubsuit$).

Conversely, for a given trace $\tau_\clubsuit = (\rho_\clubsuit, \mathsf{PO}_\clubsuit, \mathsf{WR}_\clubsuit, \mathsf{WW}_\clubsuit, \mathsf{RW}_\clubsuit) \in \mathbb{T}\mathsf{r}_\mathsf{X}(\mathcal{P}_\clubsuit)$

of a program $\mathcal{P}_{\clubsuit}$ under a semantics $\mathsf{X}$, we define the trace $\tau = (\rho, \mathsf{PO}, \mathsf{WR}, \mathsf{WW}, \mathsf{RW})$ where every two components $t[r]$ and $t[w]$ are merged into a transaction $t \in \tau$. The dependency relations are defined in a straightforward way, e.g., if $(t'[w], t[w]) \in \mathsf{WW}_{\clubsuit}$ then $(t', t) \in \mathsf{WW}$.

The following lemma shows that for any semantics $\mathsf{X} \in \{\mathtt{CC}, \mathtt{PC}, \mathtt{SI}\}$, if $\tau \in \mathbb{T}\mathsf{r}_{\mathsf{X}}(\mathcal{P})$ for a program $\mathcal{P}$, then $\tau_{\clubsuit}$ is a valid trace of $\mathcal{P}_{\clubsuit}$ under $\mathsf{X}$, i.e., $\tau_{\clubsuit} \in \mathbb{T}\mathsf{r}_{\mathsf{X}}(\mathcal{P}_{\clubsuit})$. Intuitively, this lemma shows that splitting transactions in a trace and defining dependency relations appropriately cannot introduce cycles in these relations and preserves the validity of the different consistency axioms.

The proof of this lemma relies on constructing a causal order $\mathsf{CO}_{\clubsuit}$ and an arbitration order $ARB_{\clubsuit}$ for the trace $\tau_{\clubsuit}$ starting from the analogous relations in $\tau$. In the case of $\mathtt{CC}$, these are the smallest transitive relations such that:

- $\mathsf{PO}_{\clubsuit} \subseteq \mathsf{CO}_{\clubsuit} \subseteq ARB_{\clubsuit}$, and
- if $(t_1, t_2) \in \mathsf{CO}$ then $(t_1[w], t_2[r]) \in \mathsf{CO}_{\clubsuit}$, and if $(t_1, t_2) \in ARB$ then $(t_1[w], t_2[r]) \in ARB_{\clubsuit}$.

For $\mathtt{PC}$ and $\mathtt{SI}$, $\mathsf{CO}_{\clubsuit}$ must additionally satisfy: if $(t_1, t_2) \in ARB$, then $(t_1[w], t_2[w]) \in \mathsf{CO}_{\clubsuit}$. This is required in order to satisfy the axiom AxPrefix, i.e., $ARB_{\clubsuit}; \mathsf{CO}_{\clubsuit} \subset \mathsf{CO}_{\clubsuit}$, when $(t_1[w], t_2[r]) \in ARB_{\clubsuit}$ and $(t_2[r], t_2[w]) \in \mathsf{CO}_{\clubsuit}$.

This construction ensures that $\mathsf{CO}_{\clubsuit}$ is a partial order and $ARB_{\clubsuit}$ is a total order because $\mathsf{CO}$ is a partial order and $ARB$ is a total order. Also, based on the above rules, we have that: if $(t_1[w], t_2[r]) \in \mathsf{CO}_{\clubsuit}$ then $(t_1, t_2) \in \mathsf{CO}$, and similarly, if $(t_1[w], t_2[r]) \in ARB_{\clubsuit}$ then $(t_1, t_2) \in ARB$.

**Lemma 1.** *If $\tau \in \mathbb{T}r_{X}(\mathcal{P})$, then $\tau_{\clubsuit} \in \mathbb{T}r_{X}(\mathcal{P}_{\clubsuit})$.*

Before presenting a strengthening of Lemma 1 when $\mathsf{X}$ is $\mathtt{CC}$, we give an important characterization of $\mathtt{CC}$ traces. This characterization is stated in terms of acyclicity properties.

**Lemma 2.** *$\tau$ is a trace under* $\mathtt{CC}$ *iff $ARB_0^+$ and $\mathsf{CO}_0^+; \mathsf{RW}$ are acyclic ($ARB_0$ and $\mathsf{CO}_0$ are defined in Table 1).*

Next we show that a trace $\tau$ of a program $\mathcal{P}$ is $\mathtt{CC}$ iff the corresponding trace $\tau_{\clubsuit}$ of $\mathcal{P}_{\clubsuit}$ is $\mathtt{CC}$ as well. This result is based on the observation that cycles in $ARB_0^+$ or $\mathsf{CO}_0^+; \mathsf{RW}$ cannot be broken by splitting transactions.

**Lemma 3.** *A trace $\tau$ of $\mathcal{P}$ is* $\mathtt{CC}$ *iff the corresponding trace $\tau_{\clubsuit}$ of $\mathcal{P}_{\clubsuit}$ is* $\mathtt{CC}$.

The following lemma shows that a trace $\tau$ is $\mathtt{PC}$ iff the corresponding trace $\tau_{\clubsuit}$ is $\mathtt{SER}$. The if direction in the proof is based on constructing a causal order $\mathsf{CO}$ and an arbitration order $ARB$ for the trace $\tau$ from the arbitration order $ARB_{\clubsuit}$ in $\tau_{\clubsuit}$ (since $\tau_{\clubsuit}$ is a trace under serializability $\mathsf{CO}_{\clubsuit}$ and $ARB_{\clubsuit}$ coincide). These are the smallest transitive relations such that:

- if $(t_1[w], t_2[r]) \in ARB_{\clubsuit}$ then $(t_1, t_2) \in \mathsf{CO}$,
- if $(t_1[w], t_2[w]) \in ARB_{\clubsuit}$ then $(t_1, t_2) \in ARB$[6].

[6] If $t_1[w]$ is empty ($t_1$ is read-only), then we set $(t_1, t_2) \in ARB$ if $(t_1[r], t_2[w]) \in \mathsf{CO}_{\clubsuit}$. If $t_2[w]$ is empty, then $(t_1, t_2) \in ARB$ if $(t_1[w], t_2[r]) \in \mathsf{CO}_{\clubsuit}$. If both $t_1[w]$ and $t_2[w]$ are empty, then $(t_1, t_2) \in ARB$ if $(t_1[r], t_2[r]) \in \mathsf{CO}_{\clubsuit}$.

The only-if direction is based on the fact that any cycle in the dependency relations of $\tau$ that is admitted under PC (characterized in Lemma 7) is "broken" by splitting transactions. Also, splitting transactions cannot introduce new cycles that do not originate in $\tau$.

**Lemma 4.** *A trace* $\tau$ *is* PC *iff* $\tau_{\clubsuit}$ *is* SER

The lemmas above are used to prove Theorem 1 as follows:

Proof of Theorem 1: For the if direction, assume by contradiction that $\mathcal{P}$ is not robust against CC relative to PC. Then, there must exist a trace $\tau \in \mathbb{T}\mathsf{r}_{\mathtt{CC}}(\mathcal{P}) \setminus \mathbb{T}\mathsf{r}_{\mathtt{PC}}(\mathcal{P})$. Lemmas 3 and 4 imply that the corresponding trace $\tau_{\clubsuit}$ of $\mathcal{P}_{\clubsuit}$ is CC and not SER. Thus, $\mathcal{P}_{\clubsuit}$ is not robust against CC relative to SER. The only-if direction is proved similarly. □

Robustness against CC relative to SER has been shown to be reducible in polynomial time to the reachability problem under SER [10]. Given a program $\mathcal{P}$ and a control location $\ell$, the reachability problem under SER asks whether there exists an execution of $\mathcal{P}$ under SER that reaches $\ell$. Therefore, as a corollary of Theorem 1, we obtain the following:

**Corollary 1.** *Checking robustness against* CC *relative to* PC *is reducible to the reachability problem under* SER *in polynomial time.*

In the following we discuss the complexity of this problem in the case of finite-state programs (bounded data domain). The upper bound follows from Corollary 1 and standard results about the complexity of the reachability problem under sequential consistency, which extend to SER, with a bounded [35] or parametric number of processes [45]. For the lower bound, given an instance $(\mathcal{P}, \ell)$ of the reachability problem under sequential consistency, we construct a program $\mathcal{P}'$ where each statement $s$ of $\mathcal{P}$ is executed in a different transaction that guards[7] the execution of $s$ using a global lock (the lock can be implemented in our programming language as usual, e.g., using a busy wait loop for locking), and where reaching the location $\ell$ enables the execution of a "gadget" that corresponds to the SB program in Figure 3a. Executing each statement under a global lock ensures that every execution of $\mathcal{P}'$ under CC is serializable, and faithfully represents an execution of $\mathcal{P}$ under sequential consistency. Moreover, $\mathcal{P}$ reaches $\ell$ iff $\mathcal{P}'$ contains a robustness violation, which is due to the SB execution.

**Corollary 2.** *Checking robustness of a program with a fixed number of variables and bounded data domain against* CC *relative to* PC *is PSPACE-complete when the number of processes is bounded and EXPSPACE-complete, otherwise.*

## 5 Robustness Against PC Relative to SI

In this section, we show that checking robustness against PC relative to SI can be reduced in polynomial time to a reachability problem under the SER semantics. We reuse the program transformation from the previous section that allows to simulate PC behaviors on top of SER, and additionally, we provide a characterization of traces that distinguish the PC semantics from SI. We use this

[7] That is, the transaction is of the form [lock; $s$; unlock]

characterization to define an instrumentation (monitor) that is able to detect if a program under PC admits such traces.

We show that the happens-before cycles in a robustness violation (against PC relative to SI) must contain a WW dependency followed by a RW dependency, and they should not contain two successive RW dependencies. This follows from the fact that every happens-before cycle in a PC trace must contain either two successive RW dependencies, or a WW dependency followed by a RW dependency. Otherwise, the happens-before cycle would imply a cycle in the arbitration order. Then, any trace under PC where all its simple happens-before cycles contain two successive RW dependencies is possible under SI. For instance, the trace of the non-robust LU execution in Figure 3b contains WW dependency followed by a RW dependency and does not contain two successive RW dependencies which is disallowed SI, while the trace of the robust WS execution in Figure 3c contains two successive RW dependencies. As a first step, we prove the following theorem characterizing traces that are allowed under both PC and SI.

**Theorem 2.** *A program $\mathcal{P}$ is robust against* PC *relative to* SI *iff every happens-before cycle in a trace of $\mathcal{P}$ under* PC *contains two successive* RW *dependencies.*

Before giving the proof of the above theorem, we state several intermediate results that characterize cycles in PC or SI traces. First, we show that every PC trace in which all simple happens-before cycles contain two successive RW is also a SI trace.

**Lemma 5.** *If a trace $\tau$ is* PC *and all happens-before cycles in $\tau$ contain two successive* RW *dependencies, then $\tau$ is* SI*.*

The proof of Theorem 2 also relies on the following lemma that characterizes happens-before cycles permissible under SI.

**Lemma 6.** *[23, 13] If a trace $\tau$ is* SI*, then all its happens-before cycles must contain two successive* RW *dependencies.*

Proof of Theorem 2: For the only-if direction, if $\mathcal{P}$ is robust against PC relative to SI then every trace $\tau$ of $\mathcal{P}$ under PC is SI as well. Therefore, by Lemma 6, all cycles in $\tau$ contain two successive RW which concludes the proof of this direction. For the reverse, let $\tau$ be a trace of $\mathcal{P}$ under PC such that all its happens-before cycles contain two successive RW. Then, by Lemma 5, we have that $\tau$ is SI. Thus, every trace $\tau$ of $\mathcal{P}$ under PC is SI. □

Next, we present an important lemma that characterizes happens before cycles possible under the PC semantics. This is a strengthening of a result in [13] which shows that all happens before cycles under PC must have two successive dependencies in $\{\mathsf{RW}, \mathsf{WW}\}$ and at least one RW. We show that the two successive dependencies cannot be RW followed WW, or two successive WW.

**Lemma 7.** *If a trace $\tau$ is* PC *then all happens-before cycles in $\tau$ must contain either two successive* RW *dependencies or a* WW *dependency followed by a* RW *dependency.*

Combining the results of Theorem 2 and Lemmas 4 and 7, we obtain the following characterization of traces which violate robustness against PC relative to SI.

**Theorem 3.** *A program $\mathcal{P}$ is not robust against* PC *relative to* SI *iff there exists a trace $\tau_\clubsuit$ of $\mathcal{P}_\clubsuit$ under* SER *such that the trace $\tau$ obtained by merging*[8] *read and write transactions in $\tau_\clubsuit$ contains a happens-before cycle that does not contain two successive* RW *dependencies, and it contains a* WW *dependency followed by a* RW *dependency.*

The results above enable a reduction from checking robustness against PC relative to SI to a reachability problem under the SER semantics. For a program $\mathcal{P}$, we define an instrumentation denoted by $[\![\mathcal{P}]\!]$, such that $\mathcal{P}$ is not robust against PC relative to SI iff $[\![\mathcal{P}]\!]$ violates an assertion under SER. The instrumentation consists in rewriting every transaction of $\mathcal{P}$ as shown in Figure 6.

The instrumentation $[\![\mathcal{P}]\!]$ running under SER simulates the PC semantics of $\mathcal{P}$ using the same idea of decoupling the execution of the read part of a transaction from the write part. It violates an assertion when it simulates a PC trace containing a happens-before cycle as in Theorem 3. The execution corresponding to this trace has the shape given in Figure 5, where $t_\#$ is the transaction that occurs between the WW and the RW dependencies, and every transaction executed after $t_\#$ (this can be a full transaction in $\mathcal{P}$, or only the read or write part of a transaction in $\mathcal{P}$) is related by a happens-before path to $t_\#$ (otherwise, the execution of this transaction can be reordered to occur before $t_\#$). A transaction in $\mathcal{P}$ can have its read part included in $\alpha$ and the write part included in $\beta$ or $\gamma$. Also, $\beta$ and $\gamma$ may contain transactions in $\mathcal{P}$ that executed only their read part. It is possible that $t_0 = t$, $\beta = \gamma = \epsilon$, and $\alpha = \epsilon$ (the LU program shown in Figure 3b is an example where this can happen). The instrumentation uses auxiliary variables to track happens-before dependencies, which are explained below.

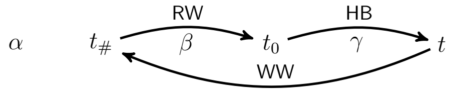

Fig. 5: Execution simulating a violation to robustness against PC relative to SI.

The instrumentation executes (incomplete) transactions without affecting the auxiliary variables (without tracking happens-before dependencies) (lines 3 and 5) until a non-deterministically chosen point in time when it declares the current transaction as the candidate for $t_\#$ (line 9). Only one candidate for $t_\#$ can be chosen during the execution. This transaction executes only its reads and it chooses non-deterministically a variable that it could write as a witness for the WW dependency (see lines 16-22). The name of this variable is stored in a global variable `varW` (see the definition of $\mathcal{I}_\#($ x := e $)$). The writes are *not* applied on the shared memory. Intuitively, $t_\#$ should be thought as a transaction whose writes are delayed for later, after transaction $t$ in Figure 5 executed. The instrumentation checks that $t_\#$ and $t$ can be connected by some happens-before path that includes the RW and WW dependencies, and that does not contain two consecutive RW dependencies. If it is the case, it violates an assertion at the commit point of $t$. Since the write part of $t_\#$ is intuitively delayed to execute after $t$, the process executing $t_\#$ is disabled all along the execution (see the `assume false`).

[8] This transformation has been defined at the beginning of Section 4.

Transaction "**begin** ⟨read⟩* ⟨test⟩* ⟨write⟩* **commit**" is rewritten to:

```
1  if ( !done# )
2   if ( * )
3    begin <read>* <test>* commit
4    if ( !done# )
5     begin <write>* commit
6    else
7     𝓘(begin) (𝓘(<write>))* 𝓘(commit)
8   else
9    begin (𝓘#(<read>))* <test>* (𝓘#(<write>))* 𝓘#(commit)
10   assume false;
11 else if ( * )
12  rdSet' := ∅;
13  wrSet' := ∅;
14  𝓘(begin) (𝓘(<read>))* <test>* 𝓘(commit)
15  𝓘(begin) (𝓘(<write>))* 𝓘(commit)
```

$\mathcal{I}_\#$( r := x ):

```
16 r := x;
17 hbR['x'] := 0;
18 rdSet := rdSet ∪ { 'x' };
```

$\mathcal{I}_\#$( x := e ):

```
19 if ( varW == ⊥ and * )
20   varW := 'x';
```

$\mathcal{I}_\#$( commit ):

```
21 assume ( varW != ⊥ )
22 done# := true
```

$\mathcal{I}$( begin ):

```
23 begin
24 hb := ⊥
25 if ( hbP != ⊥ and hbP < 2 )
26   hb := 0;
27 else if ( hbP = 2 )
28   hb := 2;
```

$\mathcal{I}$( commit ):

```
29 assume ( hb != ⊥ )
30 assert ( hb == 2 or varW ∉ wrSet' );
31 if ( hbP == ⊥ or hbP > hb )
32   hbP = hb;
33 for each 'x' ∈ wrSet'
34   if ( hbW['x'] == ⊥ or hbW['x'] > hb )
35     hbW['x'] = hb;
36 for each 'x' ∈ rdSet'
37   if ( hbR['x'] == ⊥ or hbR['x'] > hb )
38     hbR['x'] = hb;
39 rdSet := rdSet ∪ rdSet';
40 wrSet := wrSet ∪ wrSet';
41 commit
```

$\mathcal{I}$( r := x ):

```
42 r := x;
43 rdSet' := rdSet' ∪ { 'x' };
44 if ( 'x' ∈ wrSet )
45   if ( hbW['x'] != 2 )
46     hb := 0
47   else if ( hb == ⊥ )
48     hb := hbW['x']
```

$\mathcal{I}$( x := e ):

```
49 x := e;
50 wrSet' := wrSet' ∪ { 'x' };
51 if ( 'x' ∈ wrSet )
52   if ( hbW['x'] != 2 )
53     hb := 0
54   else if ( hb == ⊥ )
55     hb := hbW['x']
56 if ( 'x' ∈ rdSet )
57   if ( hb = ⊥ or hb > hbR['x'] + 1 )
58     hb := min(hbR['x'] + 1,2)
```

Fig. 6: A program instrumentation for checking robustness against `PC` relative to `SI`. The auxiliary variables used by the instrumentation are shared variables, except for `hbP`, `rdSet'`, and `wrSet'`, which are process-local variables, and they are initially set to ⊥. This instrumentation uses program constructs which can be defined as syntactic sugar from the syntax presented in Section 3, e.g., if-then-else statements (outside transactions).

After choosing the candidate for $t_\#$, the instrumentation uses the auxiliary variables for tracking happens-before dependencies. Therefore, `rdSet` and `wrSet` record variables read and written, respectively, by transactions that are connected by a happens-before path to $t_\#$ (in a trace of $\mathcal{P}$). This is ensured by the assume at line 29. During the execution, the variables read or written by a transaction[9] that writes a variable in `rdSet` (see line 56), or reads or writes a variable in `wrSet` (see lines 44 and 51), will be added to these sets (see lines 39

[9] These are stored in the local variables `rdSet'` and `wrSet'` while the transaction is running.

and 40). Since the variables that $t_{\#}$ writes in $\mathcal{P}$ are not recorded in `wrSet`, these happens-before paths must necessarily start with a RW dependency (from $t_{\#}$). When the assertion fails (line 30), the condition `varW` $\in$ `wrSet`' ensures that the current transaction has a WW dependency towards the write part of $t_{\#}$ (the current transaction plays the role of $t$ in Figure 5).

The rest of the instrumentation checks that there exists a happens-before path from $t_{\#}$ to $t$ that does not include two consecutive RW dependencies, called a $\mathtt{SI}_{\neg}$ path. This check is based on the auxiliary variables whose name is prefixed by `hb` and which take values in the domain $\{\bot, 0, 1, 2\}$ ($\bot$ represents the initial value). Therefore,

– `hbR['x']` (resp., `hbW['x']`) is 0 iff there exists a transaction $t'$ that reads `x` (resp., writes to `x`), such that there exists a $\mathtt{SI}_{\neg}$ path from $t_{\#}$ to $t'$ that ends with a dependency which is *not* RW,
– `hbR['x']` (resp., `hbW['x']`) is 1 iff there exists a transaction $t'$ that reads `x` (resp., writes to `x`) that is connected to $t_{\#}$ by a $\mathtt{SI}_{\neg}$ path, and *every* $\mathtt{SI}_{\neg}$ path from $t_{\#}$ to a transaction $t''$ that reads `x` (resp., writes to `x`) ends with an RW dependency,
– `hbR['x']` (resp., `hbW['x']`) is 2 iff there exists no $\mathtt{SI}_{\neg}$ path from $t_{\#}$ to a transaction $t'$ that reads `x` (resp., writes to `x`).

The local variable `hbP` has the same interpretation, except that $t'$ and $t''$ are instantiated over transactions in the same process (that already executed) instead of transactions that read or write a certain variable. Similarly, the variable `hb` is a particular case where $t'$ and $t''$ are instantiated to the current transaction. The violation of the assertion at line 30 implies that `hb` is 0 or 1, which means that there exists a $\mathtt{SI}_{\neg}$ path from $t_{\#}$ to $t$.

During each transaction that executes after $t_{\#}$, the variable `hb` characterizing happens-before paths that end in this transaction is updated every time a new happens-before dependency is witnessed (using the values of the other variables). For instance, when witnessing a WR dependency (line 44), if there exists a $\mathtt{SI}_{\neg}$ path to a transaction that writes to `x`, then the path that continues with the WR dependency towards the current transaction is also a $\mathtt{SI}_{\neg}$ path, and the last dependency of this path is not RW. Therefore, `hb` is set to 0 (see line 46). Otherwise, if every path to a transaction that writes to `x` is not a $\mathtt{SI}_{\neg}$ path, then every path that continues to the current transaction (by taking the WR dependency) remains a non $\mathtt{SI}_{\neg}$ path, and `hb` is set to the value of `hbW['x']`, which is 2 in this case (see line 48). Before ending a transaction, the value of `hb` can be used to modify the `hbR`, `hbW`, and `hbP` variables, but only if those variables contain bigger values (see lines 31–38).

The correctness of the instrumentation is stated in the following theorem.

**Theorem 4.** *A program $\mathcal{P}$ is robust against* PC *relative to* SI *iff the instrumentation in Figure 6 does not violate an assertion when executed under* SER.

Theorem 4 implies the following complexity result for finite-state programs. The lower bound is proved similarly to the case CC vs PC.

**Corollary 3.** *Checking robustness of a program with a fixed number of variables and bounded data domain against* PC *relative to* SI *is PSPACE-complete when the number of processes is bounded and EXPSPACE-complete, otherwise.*

Checking robustness against CC relative to SI can be also shown to be reducible (in polynomial time) to a reachability problem under SER by combining the results of checking robustness against CC relative to PC and PC relative to SI.

**Theorem 5.** *A program $\mathcal{P}$ is robust against* CC *relative to* SI *iff $\mathcal{P}$ is robust against* CC *relative to* PC *and $\mathcal{P}$ is robust against* PC *relative to* SI*.*

*Remark 1.* Our reductions of robustness checking to reachability apply to an extension of our programming language where the number of processes is unbounded and each process can execute an arbitrary number of times a statically known set of transactions. This holds because the instrumentation in Figure 6 and the one in [10] (for the case CC vs. SER) consist in adding a set of instructions that manipulate a fixed set of process-local or shared variables, which do not store process or transaction identifiers. These reductions extend also to SQL queries that access unbounded size tables. Rows in a table can be interpreted as memory locations (identified by primary keys in unbounded domains, e.g., integers), and SQL queries can be interpreted as instructions that read/write a set of locations in one shot. These possibly unbounded sets of locations can be represented symbolically using the conditions in the SQL queries (e.g., the condition in the WHERE part of a SELECT). The instrumentation in Figure 6 needs to be adapted so that read and write sets are updated by adding sets of locations for a given instruction (represented symbolically as mentioned above).

## 6 Proving Robustness Using Commutativity Dependency Graphs

We describe an approximated technique for proving robustness, which leverages the concept of left/right mover in Lipton's reduction theory [39]. This technique reasons on the *commutativity dependency graph* [9] associated to the transformation $\mathcal{P}_\clubsuit$ of an input program $\mathcal{P}$ that allows to simulate the PC semantics under serializability (we use a slight variation of the original definition of this class of graphs). We characterize robustness against CC relative to PC and PC relative to SI in terms of certain properties that (simple) cycles in this graph must satisfy.

We recall the concept of movers and the definition of commutativity dependency graphs. Given a program $\mathcal{P}$ and a trace $\tau = t_1 \cdot \ldots \cdot t_n \in \mathbb{T}\mathsf{r}_{\mathtt{SER}}(\mathcal{P})$ of $\mathcal{P}$ under serializability, we say that $t_i \in \tau$ *moves right (resp., left)* in $\tau$ if $t_1 \cdot \ldots \cdot t_{i-1} \cdot t_{i+1} \cdot t_i \cdot t_{i+2} \cdot \ldots \cdot t_n$ (resp., $t_1 \cdot \ldots \cdot t_{i-2} \cdot t_i \cdot t_{i-1} \cdot t_{i+1} \cdot \ldots \cdot t_n$) is also a valid execution of $\mathcal{P}$, $t_i$ and $t_{i+1}$ (resp., $t_{i-1}$) are executed by distinct processes, and both traces reach the same end state. A transaction $t \in \mathsf{Tr}(\mathcal{P})$ is not a right (resp., left) mover iff there exists a trace $\tau \in \mathbb{T}\mathsf{r}_{\mathtt{SER}}(\mathcal{P})$ such that $t \in \tau$ and $t$ doesn't move right (resp., left) in $\tau$. Thus, when a transaction $t$ is *not* a right mover then there must exist another transaction $t' \in \tau$ which caused $t$ to not be permutable to the right (while preserving the end state). Since $t$ and $t'$ do not commute, then this must be because of either a write-read, write-write, or a read-write dependency relation between the two transactions. We say that $t$ is not a right mover because of $t'$ and a dependency relation that is either write-read, write-write, or read-write. Notice that when $t$ is not a right mover because of $t'$ then $t'$ is not a left mover because of $t$.

We define $\mathsf{M_{WR}}$ as a binary relation between transactions such that $(t, t') \in \mathsf{M_{WR}}$ when $t$ is *not* a right mover because of $t'$ and a write-read dependency ($t'$ reads some value written by $t$). We define the relations $\mathsf{M_{WW}}$ and $\mathsf{M_{RW}}$ corresponding to write-write and read-write dependencies in a similar way. We call $\mathsf{M_{WR}}$, $\mathsf{M_{WW}}$, and $\mathsf{M_{RW}}$, *non-mover* relations.

The *commutativity dependency graph* of a program $\mathcal{P}$ is a graph where vertices represent transactions in $\mathcal{P}$. Two vertices are linked by a program order edge if the two transactions are executed by the same process. The other edges in this graph represent the "non-mover" relations $\mathsf{M_{WR}}$, $\mathsf{M_{WW}}$, and $\mathsf{M_{RW}}$. Two vertices that represent the two components $t[w]$ and $t[r]$ of the same transaction $t$ (already linked by $\mathsf{PO}$ edge) are also linked by an undirected edge labeled by $\mathsf{STO}$ (same-transaction relation).

Our results about the robustness of a program $\mathcal{P}$ are stated over a slight variation of the commutativity dependency graph of $\mathcal{P}_\clubsuit$ (where a transaction is either read-only or write-only). This graph contains additional undirected edges that link every pair of transactions $t[r]$ and $t[w]$ of $\mathcal{P}_\clubsuit$ that were originally components of the same transaction $t$ in $\mathcal{P}$. Given such a commutativity dependency graph, the robustness of $\mathcal{P}$ is implied by the absence of cycles of specific shapes. These cycles can be seen as an abstraction of potential robustness violations for the respective semantics (see Theorem 6 and Theorem 7). Figure 7 pictures the commutativity dependency graph for the $\mathsf{MP}$ program. Since every transaction in $\mathsf{MP}$ is singleton, the two programs $\mathsf{MP}$ and $\mathsf{MP}_\clubsuit$ coincide.

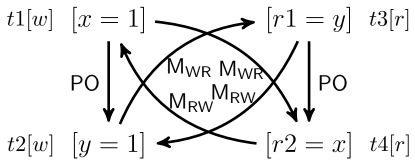

Fig. 7: The commutativity dependency graph of the $\mathsf{MP}_\clubsuit$ program.

Using the characterization of robustness violations against CC relative to SER from [10] and the reduction in Theorem 1, we obtain the following result concerning the robustness against CC relative to PC.

**Theorem 6.** *Given a program $\mathcal{P}$, if the commutativity dependency graph of the program $\mathcal{P}_\clubsuit$ does not contain a simple cycle formed by $t_1 \cdots t_i \cdots t_n$ such that:*

- $(t_n, t_1) \in \mathsf{M_{RW}}$*;*
- $(t_j, t_{j+1}) \in (\mathsf{PO} \cup \mathsf{WR})^*$*, for* $j \in [1, i-1]$*;*
- $(t_i, t_{i+1}) \in (\mathsf{M_{RW}} \cup \mathsf{M_{WW}})$*;*
- $(t_j, t_{j+1}) \in (\mathsf{M_{RW}} \cup \mathsf{M_{WW}} \cup \mathsf{M_{WR}} \cup \mathsf{PO})$*, for* $j \in [i+1, n-1]$*.*

*then $\mathcal{P}$ is robust against* CC *relative to* PC*.*

Next we give the characterization of commutativity dependency graphs required for proving robustness against PC relative to SI.

**Theorem 7.** *Given a program $\mathcal{P}$, if the commutativity dependency graph of the program $\mathcal{P}_{\clubsuit}$ does not contain a simple cycle formed by $t_1 \cdots t_n$ such that:*

- $(t_n, t_1) \in \mathsf{M_{WW}}$, $(t_1, t_2) \in \mathsf{STO}$, *and* $(t_2, t_3) \in \mathsf{M_{RW}}$*;*
- $(t_j, t_{j+1}) \in (\mathsf{M_{RW}} \cup \mathsf{M_{WW}} \cup \mathsf{M_{WR}} \cup \mathsf{PO} \cup \mathsf{STO})^*$, *for* $j \in [3, n-1]$*;*
- $\forall\ j \in [2, n-2]$.
  - *if* $(t_j, t_{j+1}) \in \mathsf{M_{RW}}$ *then* $(t_{j+1}, t_{j+2}) \in (\mathsf{M_{WR}} \cup \mathsf{PO} \cup \mathsf{M_{WW}})$*;*
  - *if* $(t_{j+1}, t_{j+2}) \in \mathsf{M_{RW}}$ *then* $(t_j, t_{j+1}) \in (\mathsf{M_{WR}} \cup \mathsf{PO})$.
- $\forall\ j \in [3, n-3]$. *if* $(t_{j+1}, t_{j+2}) \in \mathsf{STO}$ *and* $(t_{j+2}, t_{j+3}) \in \mathsf{M_{RW}}$ *then* $(t_j, t_{j+1}) \in \mathsf{M_{WW}}$.

*then* $\mathcal{P}$ *is robust against* `PC` *relative to* `SI`.

In Figure 7, we have three simple cycles in the graph:

- $(t1[w], t4[r]) \in \mathsf{M_{WR}}$ and $(t4[r], t1[w]) \in \mathsf{M_{RW}}$,
- $(t2[w], t3[r]) \in \mathsf{M_{WR}}$ and $(t3[r], t2[w]) \in \mathsf{M_{RW}}$,
- $(t1[w], t2[w]) \in \mathsf{PO}$, $(t2[w], t3[r]) \in \mathsf{M_{WR}}$, $(t3[r], t4[r]) \in \mathsf{PO}$, and $(t4[r], t1[w]) \in \mathsf{M_{RW}}$.

Notice that none of the cycles satisfies the properties in Theorems 6 and 7. Therefore, MP is robust against `CC` relative to `PC` and against `PC` relative to `SI`.

*Remark 2.* For programs that contain an unbounded number of processes, an unbounded number of instantiations of a fixed number of process "templates", or unbounded loops with bodies that contain entire transactions, a sound robustness check consists in applying Theorem 6 and Theorem 7 to (bounded) programs that contain two copies of each process template, and where each loop is unfolded exactly two times. This holds because the mover relations are "static", they do not depend on the context in which the transactions execute, and each cycle requiring more than two process instances or more than two loop iterations can be short-circuited to a cycle that exists also in the bounded program. Every outgoing edge from a third instance/iteration can also be taken from the second instance/iteration. Two copies/iterations are necessary in order to discover cycles between instances of the same transaction (the cycles in Theorem 6 and Theorem 7 are simple and cannot contain the same transaction twice). These results extend easily to SQL queries as well because the notion of mover is independent of particular classes of programs or instructions.

## 7 Experimental Evaluation

We evaluated our approach for checking robustness on 7 applications extracted from the literature on databases and distributed systems, and an application Betting designed by ourselves. Two applications were extracted from the OLTP-Bench benchmark [30]: a vote recording application (Vote) and a consumer review application (Epinions). Three applications were obtained from Github projects (used also in [9, 19]): a distributed lock application for the Cassandra database (CassandraLock [24]), an application for recording trade activities

(SimpleCurrencyExchange [48]), and a micro social media application (Twitter [49]). The last two applications are a movie ticketing application (FusionTicket) [34], and a user subscription application inspired by the Twitter application (Subscription). Each application consists of a set of SQL transactions that can be called an arbitrary number of times from an arbitrary number of processes. For instance, Subscription provides an AddUser transaction for adding a new user with a given username and password, and a RemoveUser transaction for removing an existing user. (The examples in Figure 1 are particular variations of FusionTicket, Twitter, and Betting.) We considered five variations of the robustness problem: the three robustness problems we studied in this paper along with robustness against SI relative to SER and against CC relative to SER. The artifacts are available in a GitHub repository [31].

Table 2: Results of the experiments. The columns titled X-Y stand for the result of applications robustness against X relative to Y.

| Application | Transactions | Robustness | | | | |
|---|---|---|---|---|---|---|
| | | CC-PC | PC-SI | CC-SI | SI-SER | CC-SER |
| Betting | 2 | yes | yes | yes | yes | yes |
| CassandraLock | 3 | yes | yes | yes | yes | yes |
| Epinions | 8 | no | yes | no | yes | no |
| FusionTicket | 3 | no | no | no | yes | no |
| SimpleCurrencyExchange | 4 | yes | yes | yes | yes | yes |
| Subscription | 2 | yes | no | no | yes | no |
| Twitter | 3 | no | no | no | yes | no |
| Vote | 1 | yes | yes | yes | no | no |

In the first part of the experiments, we check for robustness violations in bounded-size executions of a given application. For each application, we have constructed a client program with a fixed number of processes (2) and a fixed number of transactions of the corresponding application (at most 2 transactions per process). For each program and pair of consistency models, we check for robustness violations using the reductions to reachability under SER presented in Section 4 and Section 5 in the case of pairs of weak consistency models, and the reductions in [9, 10] when checking for robustness relative to SER.

We check for reachability (assertion violations) using the Boogie program verifier [8]. We model tables as unbounded maps in Boogie and SQL queries as first-order formulas over these maps (that may contain existential or universal quantifiers). To model the uniqueness of primary keys we use Boogie linear types.

Table 2 reports the results of this experiment (cells filled with "no")[10]. Five applications are not robust against at least one of the semantics relative to some other stronger semantics. The runtimes (wall-clock times) for the robustness checks are all under one second, and the memory consumption is around 50 Megabytes. Concerning scalability, the reductions to reachability presented in Section 4 and Section 5 show that checking robustness is as hard as checking

[10] The Twitter client in Table 2, which is not PC vs CC robust, is different from the one described in Section 2. This client program consists of two processes, each executing FollowUser and AddTweet.

reachability (the size of the instrumented program is only linear in the size of the original program). Therefore, checking robustness will also suffer from the classic state explosion problem when increasing the number of processes. On the other hand, increasing the number of transactions in a process does not seem to introduce a large overhead. Increasing the number of transactions per process in the clients of Epinions, FusionTicket, and SimpleCurrencyExchange from 2 to 5 introduces a running time overhead of at most 25%.

All the robustness violations we report correspond to violations of the intended specifications. For instance: (1) the robustness violation of Epinions against CC relative to PC allows two users to update their ratings for a given product and then when each user queries the overall rating of this product they do not observe the latest rating that was given by the other user, (2) the robustness violation of Subscription against PC relative to SI allows two users to register new accounts with the same identifier, and (3) the robustness violation of Vote against SI relative to SER allows the same user to vote twice. The specification violation in Twitter was reported in [19]. However, it was reported as violation of a different robustness property (CC relative to SER) while our work shows that the violation persists when replacing a weak consistency model (e.g., SI) with a weaker one (e.g. CC). This implies that this specification violation is not present under SI (since it appears in the difference between CC and SI behaviors), which cannot be deduced from previous work.

In the second part of the experiments, we used the technique described in Section 6, based on commutativity dependency graphs, to prove robustness. For each application (set of transactions) we considered a program that for each ordered pair of (possibly identical) transactions in the application, contains two processes executing that pair of transactions. Following Remark 2, the robustness of such a program implies the robustness of a *most general client* of the application that executes each transaction an arbitrary number of times and from an arbitrary number of processes. We focused on the cases where we could not find robustness violations in the first part. To build the "non-mover" relations $\mathsf{M}_{\mathsf{WR}}$, $\mathsf{M}_{\mathsf{WW}}$, and $\mathsf{M}_{\mathsf{RW}}$ for the commutativity dependency graph, we use the left/right mover check provided by the CIVL verifier [33]. The results are reported in Table 2, the cells filled with "yes". We showed that the three applications Betting, CassandraLock and SimpleCurrencyExchange are robust against any semantics relative to some other stronger semantics. As mentioned earlier, all these robustness results are established for arbitrarily large executions and clients with an arbitrary number of processes. For instance, the robustness of SimpleCurrencyExchange ensures that when the exchange market owner observes a trade registered by a user, they observe also all the other trades that were done by this user in the past.

In conclusion, our experiments show that the robustness checking techniques we present are effective in proving or disproving robustness of concrete applications. Moreover, it shows that the robustness property for different combinations of consistency models is a relevant design principle, that can help in choosing the right consistency model for realistic applications, i.e., navigating the tradeoff between consistency and performance (in general, weakening the consistency leads to better performance).

## 8 Related Work

The consistency models in this paper were studied in several recent works [21, 20, 25, 43, 16, 44, 14]. Most of them focused on their operational and axiomatic formalizations. The formal definitions we use in this paper are based on those given in [25, 16]. Biswas and Enea [14] shows that checking whether an execution is CC is polynomial time while checking whether it is PC or SI is NP-complete.

The robustness problem we study in this paper has been investigated in the context of weak memory models, but only relative to sequential consistency, against Release/Aquire (RA), TSO and Power [36, 17, 15, 29]. Checking robustness against CC and SI relative to SER has been investigated in [9, 10]. In this work, we study the robustness problem between two weak consistency models, which poses different non-trivial challenges. In particular, previous work proposed reductions to reachability under sequential consistency (or SER) that relied on a concept of minimal robustness violations (w.r.t. an operational semantics), which does not apply in our case. The relationship between PC and SER is similar in spirit to the one given by Biswas and Enea [14] in the context of checking whether an execution is PC. However, that relationship was proven in the context of a "weaker" notion of trace (containing only program order and read-from), and it does not extend to our notion of trace. For instance, that result does not imply preserving WW dependencies which is crucial in our case.

Some works describe various over- or under-approximate analyses for checking robustness relative to SER. The works in [13, 18, 19, 26, 40] propose static analysis techniques based on computing an abstraction of the set of computations, which is used for proving robustness. In particular, [19, 40] encode program executions under the weak consistency model using FOL formulas to describe the dependency relations between actions in the executions. These approaches may return false alarms due to the abstractions they consider in their encoding. Note that in this paper, we prove a strengthening of the results of [13] with regard to the shape of happens before cycles allowed under PC.

An alternative to *trace-based* robustness, is *state-based* robustness which requires that a program is robust if the sets of reachable states under two semantics coincide. While state-robustness is the necessary and sufficient concept for preserving state-invariants, its verification, which amounts in computing the set of reachable states under the weak semantics models is in general a hard problem. The decidability and the complexity of this problem has been investigated in the context of relaxed memory models such as TSO and Power, and it has been shown that it is either decidable but highly complex (non-primitive recursive), or undecidable [5, 6]. Automatic procedures for approximate reachability/invariant checking have been proposed using either abstractions or bounded analyses, e.g., [7, 4, 28, 1]. Proof methods have also been developed for verifying invariants in the context of weakly consistent models such as [37, 32, 41, 3]. These methods, however, do not provide decision procedures.
